# RNA Interference of Gonadotropin-Inhibitory Hormone Gene Induces Arousal in Songbirds

**DOI:** 10.1371/journal.pone.0030202

**Published:** 2012-01-18

**Authors:** Takayoshi Ubuka, Motoko Mukai, Jordan Wolfe, Ryan Beverly, Sarah Clegg, Ariel Wang, Serena Hsia, Molly Li, Jesse S. Krause, Takanobu Mizuno, Yujiro Fukuda, Kazuyoshi Tsutsui, George E. Bentley, John C. Wingfield

**Affiliations:** 1 Department of Integrative Biology and Helen Wills Neuroscience Institute, University of California, Berkeley, California, United States of America; 2 Department of Neurobiology, Physiology and Behavior, University of California Davis, Davis, California, United States of America; 3 Department of Biology, Waseda University, Shinjuku, Tokyo, Japan; 4 Department of Population Medicine and Diagnostic Sciences, College of Veterinary Medicine, Cornell University, Ithaca, New York, United States of America; Nagoya University, Japan

## Abstract

Gonadotropin-inhibitory hormone (GnIH) was originally identified in quail as a hypothalamic neuropeptide inhibitor of pituitary gonadotropin synthesis and release. However, GnIH neuronal fibers do not only terminate in the median eminence to control anterior pituitary function but also extend widely in the brain, suggesting it has multiple roles in the regulation of behavior. To identify the role of GnIH neurons in the regulation of behavior, we investigated the effect of RNA interference (RNAi) of the GnIH gene on the behavior of white-crowned sparrows, a highly social songbird species. Administration of small interfering RNA against GnIH precursor mRNA into the third ventricle of male and female birds reduced resting time, spontaneous production of complex vocalizations, and stimulated brief agonistic vocalizations. GnIH RNAi further enhanced song production of short duration in male birds when they were challenged by playbacks of novel male songs. These behaviors resembled those of breeding birds during territorial defense. The overall results suggest that GnIH gene silencing induces arousal. In addition, the activities of male and female birds were negatively correlated with GnIH mRNA expression in the paraventricular nucleus. Density of GnIH neuronal fibers in the ventral tegmental area was decreased by GnIH RNAi treatment in female birds, and the number of gonadotropin-releasing hormone neurons that received close appositions of GnIH neuronal fiber terminals was negatively correlated with the activity of male birds. In summary, GnIH may decrease arousal level resulting in the inhibition of specific motivated behavior such as in reproductive contexts.

## Introduction

Gonadotropin-inhibitory hormone (GnIH) is a hypothalamic neuropeptide that inhibits gonadotropin secretion *in vitro* [1] and *in vivo* [2]. It is synthesized in neurons of the paraventricular nucleus (PVN) in birds [3]–[5]. GnIH neurons project to the median eminence [1], [3] and control anterior pituitary hormone release, and also to other regions of the brain suggesting the regulation of autonomic function and behavior [4]–[6]. The mRNA of its cognate G protein-coupled receptor was also found to be expressed in various parts of the brain [4], [7]. A previous study has shown that central administration of GnIH to the third ventricle of female white-crowned sparrows (*Zonotrichia leucophrys gambelii*) inhibits copulation solicitation, which is analogous to mammalian lordosis [8]. Similarly, central administration of a GnIH ortholog, RFamide-related peptide (RFRP) [9]–[12], suppressed male sexual behavior of rats [13], [14].

In order to understand the function of GnIH neurons in the brain, we tested the effect of GnIH gene silencing on the behavior of male and female white-crowned sparrows by suppressing GnIH mRNA expression with the administration of corresponding double-stranded RNA (siRNA) [15]. White-crowned sparrow is a highly social songbird species that its neuroendocrine system has been studied for decades. Accordingly, it serves as an excellent animal model to study the molecular mechanism of behavior. We hypothesized that reduction of GnIH expression will positively regulate sexual behavior, in contrast to the GnIH administration. It was recently proposed that generalized arousal of the central nervous system (CNS) underlies specific motivated behavior such as sexual behavior [16]. An animal with a greater degree of CNS arousal (*1*) shows greater responsiveness to sensory stimuli in all sensory modalities; (*2*) emits more motor activity; and (*3*) is more reactive emotionally [17]. Reduction of GnIH may lead to the generalized arousal. Therefore, we measured spontaneous behavior and responses to playbacks of novel male songs because the latter is a strong sensory stimulus to induce motivated behavior such as territorial behavior in male (*1*). We specifically measured the resting time to indirectly quantify locomotor activity (*2*), and various vocalizations as a measurement of reactive emotions of songbirds within a social context (*3*).

Our behavioral analyses revealed significant increases in spontaneous locomotor activities of male and female birds by GnIH RNAi as if the birds were challenged by playbacks of novel male songs, potentially suggesting generalized arousal of the CNS. Thus, we further analyzed the relationships between the activities of birds and GnIH mRNA expression levels in the PVN and GnIH neuronal fiber distributions. GnIH neuronal fibers terminate on gonadotropin-releasing hormone (GnRH) -I and -II neurons [18], and these neurons express GnIH receptor mRNA in songbirds [4]. Accordingly, it is possible that GnIH inhibits both sexual and generalized arousal by decreasing the activities of GnRH-I and GnRH-II neurons. We investigated this by analyzing the relationships between the activities of the birds and the numbers of GnRH-I and -II neurons with close appositions of GnIH neuronal fiber terminal like structures.

## Materials and Methods

### Experimental schedule

White-crowned sparrows were caught in Davis (California, USA) during March of 2008, and kept in a short-day light/dark cycle (9 h light/15 h dark, lights on at 8 a.m.) in individual cages. Food and water were given *ad libitum*. Brain cannulation to the third ventricle was performed according to our established method [8], [19]. On March 27^th^, all birds were photostimulated using a long-day light/dark cycle (20 h light/4 h dark, lights on at 5 a.m.). Correct insertions of the cannulae were tested by the induction of drinking behavior with angiotensin-II infusion [19]. On April 15^th^ birds were allocated either to the experimental room (8 males and 8 females) or to the control room (8 males and 8 females). Cages of male or female birds were placed alternately so that they could interact with birds of the opposite sex. On April 18^th^ 0.5 nmol of the 25 base-pair double-stranded RNA (Sense: 5’-CACAGCAAGCAAGAUGCCAAAUUCA-3’, Antisense: 5’-UGAAUUUGGCAUCUUGCUUGCUGUG-3’; Stealth; Invitrogen, Carlsbad, CA) corresponding to white-crowned sparrow GnIH precursor mRNA sequence (Genbank AB128164, nt 277–301; [20]) that does not agree with any other gene in the database was administered in the third ventricle of the experimental birds. Control birds were administered with 0.5 nmol of 25 base-pair double-stranded RNA with a sequence that does not agree with any gene in the database and has the same GC ratio as the GnIH siRNA (Sense: 5’-CACACGAACGAUACGACAAUGAUCA-3’, Antisense: 5’-UGAUCAUUGUCGUAUCGUUCGUGUG-3’Stealth Control; Invitrogen). 0.5 nmol of GnIH siRNA was estimated to be 1×10^5^ times greater than the amount of GnIH mRNA expressed in the brain. GnIH siRNA or control was infused in 5 µl sterilized 0.9% physiological saline over 1 min to each bird. Two days after the infusion of GnIH siRNA or control, spontaneous behavior were recorded by digital cameras from 9 a.m. to 11 a.m. On the same day from 11:30 a.m. to 2:30 p.m., birds were challenged with 4 different novel male song playbacks for 10 minutes each. Behavioral responses were recorded by digital cameras during each playback. On the next day, birds were euthanized using 10% isoflurane inhalation and trunk blood and brain was collected. Serum was separated and stored in −20°C and the brains were immediately frozen on dry ice and stored in −80°C until further analysis. Brains were used to analyze GnIH precursor mRNA expression and GnIH neuronal fiber distributions. In order to validate the efficacy and specificity of Stealth RNAi, GnIH concentration was measured in the diencephalon in birds that were infused with vehicle, control RNA, or GnIH RNAi. In February of 2011, 16 white-crowned sparrows (4 males, 12 females) were caught in Davis and photostimulated for three weeks using a long-day lighting cycle (20 h light/4 h dark, lights on at 5 a.m.). Birds were cannulated as described above. GnIH siRNA, control RNA, or vehicle (sterilized 0.9% physiological saline) was administered and birds were sacrificed two days after the infusion, and GnIH peptide concentration in the diencephalon was quantified. All procedures were performed in accordance with the NIH Guide for the Care and Use of Laboratory Animals and under an approved protocol from the University of California, Davis. The IACUC protocol number was 16283 with the title of ‘Control of Seasonal Breeding in Diverse Habitats’.

### Behavioral measurements

Locomotor activity of the birds was quantified indirectly by measuring the resting time of birds. Birds were regarded as resting when they were sitting still without feeding, preening, drinking, hopping or pointing. The time spent for feeding and the numbers of hopping and pointing were also measured. We also analyzed the effect of GnIH RNAi on the vocalizations of birds because this is a major portion of daily displays [21]. For example, male song is used for territorial defense, mate attraction and may stimulate nesting in the female [22]. A typical song is 2–3 seconds long with an introductory component that contains one or more continuous or segmented whistles followed by complex sounds consisting of frequency sweeps, buzzy vibrato elements, and trills. Nine distinct vocalizations other than song are identified in the white-crowned sparrow [23]. In this study, non-song vocalizations were assorted into chuckle (a group of calls used in agonistic interactions and for sexual behavior) and tseep (calls used for communication including arousal). One continuous vocalization of each type was counted as one song, chuckle and tseep. The duration of song was also quantified. Vocalizations of male and female birds that were treated with GnIH RNAi or control were analyzed together within groups because the bird which gave the vocalization was not always identified by the videotapes.

Spontaneous activities of each bird were recorded for approximately 30 minutes, and the results were expressed as the counts per 10 minutes. Four novel male songs were performed for 10 minutes each with 30 minutes’ silent period between each playback. Activities in 10 minutes were averaged from their responses to four male song playbacks. Novel male songs were recorded in the wild and played back in the room as natural as possible using an iPod (Apple, Cupertino, CA) and a large speaker. All behavioral measurements were conducted by three observers who were blind to the treatments and the results were averaged for statistical analyses.

### Measurement of GnIH precursor mRNA expression by real-time quantitative PCR

To quantify GnIH precursor mRNA expression in different brain regions, real-time quantitative PCR was conducted by using the LineGene system (LineGene FQD-33A; BioFlux, Tokyo, Japan). GADPH, a housekeeping gene, was used as an internal standard. The PCR primers used to amplify white-crowned sparrow precursor cDNA fragment were 5’-TCCAACAGCATGTGCCTAAA-3’ (identical to nucleotides 104–123; GenBank AB128164) and 5’-TTTTGATCCCCAGTCTTCCA-3’ (complementary to nucleotides 234–253). The PCR primers for GADPH were 5’-GAGGGTAGTGAAGGCTGCTG-3’ (identical to nucleotides 795–814; GenBank NM_001198610) and 5’-CAAAGGTGGAGGAATGGCTA-3’ (complementary to nucleotides 885–904; GenBank NM_001198610). The final reaction mixture contained SYBR Green Realtime PCR Master Mix (Toyobo, Osaka, Japan), 1 µM each of forward and reverse primers and 1 µg of cDNA. The PCR condition was 95°C for 2 min, followed by 40 cycles of 95°C, 20 sec; 60°C, 20 sec; 72°C, 30 sec. An external standard curve was generated by dilutions of the purified target PCR product. To confirm the specificity of the amplification, the PCR products were subjected to a melting curve analysis and gel electrophoresis. The GnIH precursor gene expression was normalized by GAPDH expression using the LineGene software (BioFlux).

### *In situ* hybridization of white-crowned sparrow GnIH precursor mRNA

Cross sections at 25 µm thickness of the white-crowned sparrow brain were collected on a cryostat at −20°C for histological studies. *In situ* hybridization was carried out with our previous method using a digoxigenin (DIG)-labeled antisense RNA probe [24]. The DIG-labeled antisense RNA probe was produced using a standard RNA labeling kit (Roche Diagnostics, Mannheim, Germany) by using partial white-crowned sparrow GnIH precursor cDNA (nt 203–511 in AB128164) as a template. Hybridized probe was visualized in blue by immersing the sections in a substrate solution (nitroblue tetrazolium/5-bromo-4-chloro-3-indolyl phosphate stock solution; Roche Diagnostics). Control for specificity of *in situ* hybridization was performed by using a DIG-labeled sense RNA probe, the sequence of which was complementary to the antisense probe. The numbers of apparently recognized GnIH precursor mRNA positive cells identified from their staining, structure and portion in the PVN were counted on the microscope at 200× by two observers who were blind to the treatments, and independent counts were averaged for statistical analysis. Microscopic images were acquired digitally on an Axio Imager, A1 microscope (Carl Zeiss AG, Gottingen, Germany) with an AxioCam MRc5 digital camera. The density of *in situ* hybridization signal of individual neuron was measured by using Sion Image (Ver. 4.02, Scion, Maryland, WA) as a gray scale value from 0 (white) to 256 (black) and expressed as the mean density per cell, which was obtained by subtracting background gray values. The product of the mean density of GnIH mRNA positive cells and the number of GnIH mRNA positive cells in three most populated sections at 100 µm rostral to caudal distances was compared between the groups. Three most populated sections of GnIH mRNA positive cells at 100 µm rostral to caudal distances from each bird were analyzed because that was the maximum number of sections to be processed simultaneously with the same working solutions for all 32 brains (n = 8 each in 4 groups), and cross sections in 275 µm (the first section of 25 µm thickness + 100 µm gap + the second section of 25 µm thickness + 100 µm gap + the third section of 25 µm thickness) rostral to caudal width contained the core of the cluster of GnIH neurons in the PVN. The number of GnIH mRNA positive cells was normalized by 1 mm square area that is vertical to rostral to caudal axis including the PVN within the hypothalamus to compare the expression of GnIH mRNA between the groups.

### Enzyme-linked immunosorbent assay (ELISA) of GnIH

Peptides were extracted from the diencephalons of 16 white-crowned sparrows (4 males, 12 females) as described [3]. Frozen diencephalic samples were boiled for 7 min and homogenized in 5% acetic acid. The homogenate was centrifuged at 16,000× g for 30 min at 4°C. The supernatant was collected and forced through a disposable C-18 cartridge (Sep-Pak Vac 1cc; Waters, Milford, MA). The retained material was then eluted with 60% methanol. The pooled eluate was concentrated in a vacuum evaporator, passed through disposable Ultrafree-MC centrifugal filter units (Millipore), and the extract was subjected to competitive ELISA by using the antiserum raised in a rabbit against GnIH [1]. In brief, different concentrations of synthetic white-crowned sparrow GnIH (SIKPFSNLPLRF-NH_2_; 1–1,000 pmol/ml) and adjusted tissue extracts were added with the antiserum against GnIH (1∶1,000 dilution) to each antigen-coated well of a 96-well microplate (multiwell plate for ELISA, H-Type; Sumitomo Bakelite, Tokyo, Japan), and incubated for 1 h at 37°C. After the reaction with alkaline phosphatase-labeled goat anti-rabbit IgG, immunoreactive products were obtained in a substrate solution of *p*-nitrophenylphosphate, and the absorbance was measured at 415 nm on a microtiter plate reader (MTP-120; Corona Electric, Ibaraki, Japan).

### Immunocytochemistry of GnIH, and GnRH

Immunocytochemical analysis of GnIH was conducted with our previous method [24] on the cross sections at 25 µm thickness. The primary antibody used was affinity column purified rabbit anti-white-crowned sparrow GnIH antibody (Pacific Immunology Corp., Ramona, CA). The specificity of the primary antibody was assessed by adsorption tests of the antibody with 1×10^−6^ M synthetic white-crowned sparrow GnIH. Immunoreactive material was visualized in purple using Vector VIP (Vector Laboratories, Burlingame, CA). Some of the sections were doubled stained using GnRH antibody according to our previous method [4], [11]. The primary antibody used to label GnRH neurons was rabbit anti-GnRH (HU60H; kindly donated by Dr. H. Urbanski, Oregon Regional Primate Center, Portland, OR). Although the antibody does not distinguish between GnRH-I (pEHWSYGLQPG-NH_2_) and GnRH-II (pEHWSHGWYPG-NH_2_) neurons, we could identify them based on their separate locations in the brain (GnRH-I neurons are located in the preoptic area, whereas GnRH-II neurons are located in the midbrain). Immunoreactive material against GnRH antibody was visualized in brown using diaminobenzidine.

Brain regions and nuclei were identified by Nissl staining of the adjacent sections using a canary brain map [25]. The density of GnIH-ir fiber was quantified by counting the number of dots of the beaded fibers in 200 µm square in the center of each brain nucleus. The number of GnRH-I, GnRH-II-ir neurons, and GnRH-I, GnRH-II-ir neurons that received putative contacts from GnIH-ir fibers were counted in the sections at 100 µm rostral to caudal distances in each brain. GnRH-I neurons which received putative contacts from GnIH-ir fibers were counted form 11.3±3.2 (mean ± SD, n = 32) sections that contained the preoptic area, whereas GnRH-II neurons which received putative contacts from GnIH-ir fibers were counted form 8.5±1.6 sections (mean ± SD, n = 32) that contained the midbrain region. A putative contact was scored only if a GnIH-ir bouton-like structure was observed in close proximity to GnRH-I or GnRH-II neurons.

### Testosterone radioimmunoassay (RIA)

Plasma testosterone concentration was measured by RIA using our previous method [26], [27].

### Statistical analysis

The effect of GnIH gene silencing on the product of the cell number and the mean density of GnIH mRNA positive cells between control and experimental birds was analyzed by two-tailed Student’s t-test. The effect of GnIH gene silencing on GnIH concentration, the resting time, the vocalizations were analyzed by one-way ANOVA followed by Fisher’s protected least significant difference (PLSD) test. Effect of GnIH gene silencing on the number of songs was further analyzed by Kruskal-Wallis test followed by Mann-Whitney U-test. The relationship between the resting time and the GnIH neuronal system, and plasma testosterone concentration was analyzed by two-sided Pearson’s correlation test. Values outside the mean ± twice standard deviation were excluded from the correlation analyses to omit the outlier which can produce a high correlation coefficient.

## Results

### Expression of GnIH precursor mRNA in the white-crowned sparrow brain

**Figure 1:**
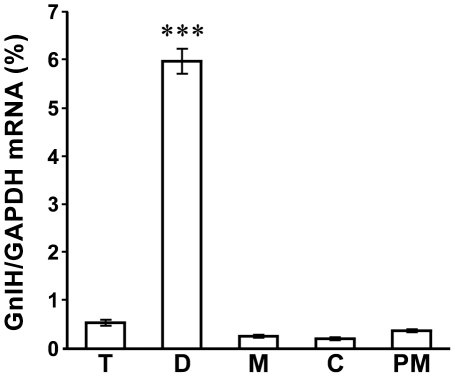
Expression of GnIH precursor mRNA in the white-crowned sparrow brain. GnIH precursor mRNA is solely expressed in the diencephalon (D) compared to other brain regions. T: telencephalon, M: midbrain, C: cerebellum, PM: pons/medulla. GnIH precursor mRNA was quantified by real-time quantitative PCR using GADPH mRNA as an internal standard. The columns and the vertical lines represent the mean ± SEM (n = 4). ***, *P*<0.001 vs. T, M, C, PM by one-way ANOVA followed by Fisher’s PLSD.

White-crowned sparrow brains of mixed sex birds (1 male, 3 female) were divided into 5 parts (T: telencephalon, D: diencephalon, M: midbrain, C: cerebellum, and PM: pons/medulla) and GnIH precursor mRNA was measured by real-time quantitative PCR. A significant amount of GnIH mRNA was detected only in the diencephalon (Fig. 1, F = 326, *P* = 4.1×10^−15^ by one-way ANOVA, *P* = 1.1×10^−14^ T vs. D, *P* = 5.2×10^−15^ D vs. M, *P* = 4.5×10^−15^ D vs. C, *P* = 7.1×10^−15^ D vs. PM by Fisher’s PLSD).

### Effect of GnIH RNAi on the expression of GnIH precursor mRNA in the PVN

**Figure 2:**
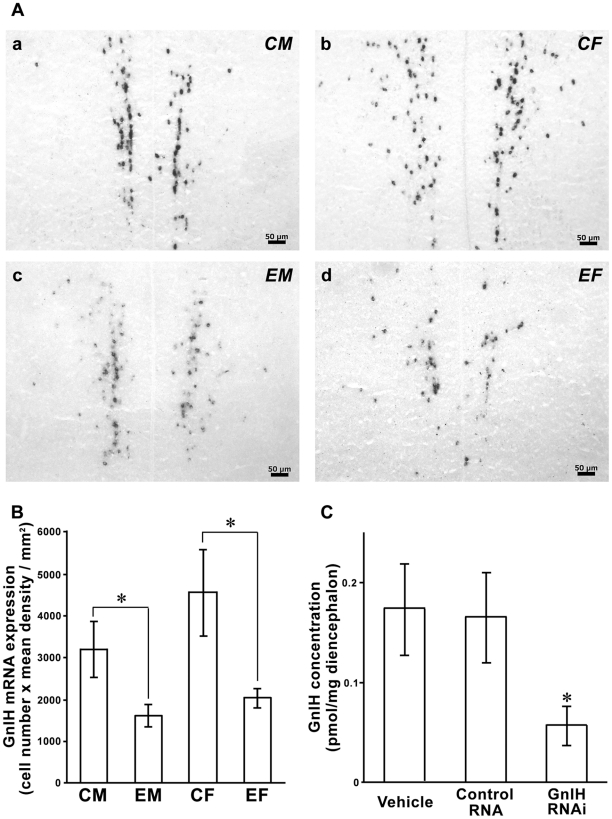
Effect of GnIH RNAi on GnIH precursor mRNA and GnIH expression. ***A,*** Expression of GnIH precursor mRNA positive cells in the paraventricular nucleus of control male (a, CM), control female (b, CF), experimental male (c, EM), and experimental female (d, EF). Bar = 50 µm. ***B,*** The product of the cell number and the mean density of *in situ* hybridization signal from three most populated sections containing GnIH precursor mRNA positive cells in CM, EM, CF, and EF bird brain. The columns and the vertical lines represent the mean ± SEM (n = 8). *, *P*<0.05 CM vs EM, CF vs EF by two-tailed Student’s *t*-test. ***C,*** Effect of GnIH RNAi on GnIH concentration in the diencephalon. GnIH concentration in the diencephalon was significantly reduced in GnIH siRNA administered birds (GnIH RNAi, n = 7) compared to vehicle (n = 4) and control RNA (n = 5) administered birds. The columns and the vertical lines represent the mean ± SEM. *, *P*<0.05 vehicle or control vs. GnIH RNAi by one-way ANOVA followed by Fisher’s PLSD.

*In situ* hybridization of GnIH precursor mRNA showed a cluster of GnIH mRNA positive cells in the PVN of hypothalamus (Fig. 2A) as per our previous study [24]. The cluster of GnIH mRNA positive cells tended to gather closer in sagittal planes on both sides of the brain in male birds (Fig. 2Aa, 2Ac) compared to female birds (Fig. 2Ab, 2Ad). The product of the cell number and the mean density of GnIH mRNA positive cells was significantly reduced in GnIH RNAi treated birds compared to the control birds both in male and female [Fig. 2B; Male, *t* = 2.2, *P* = 0.049 CM (control male) vs. EM (experimental male); Female, *t* = 2.4, *P* = 0.033 CF (control female) vs. EF (experimental female) by two-tailed Student’s *t*-test).

### Effect of GnIH RNAi on GnIH concentration in the diencephalon

GnIH concentration in the diencephalon was not reduced by control RNA administration compared to the vehicle administered birds (Fig. 2C). However, the concentration of GnIH was significantly reduced in GnIH siRNA administered birds compared to vehicle or control RNA administered birds (Fig. 2C, F = 3.93, *P* = 0.046 by one-way ANOVA, *P* = 0.036 vehicle vs. GnIH RNAi, *P* = 0.037 control RNA vs. GnIH RNAi by Fisher’s PLSD).

### Effect of GnIH RNAi on the activities of male and female birds

**Figure 3:**
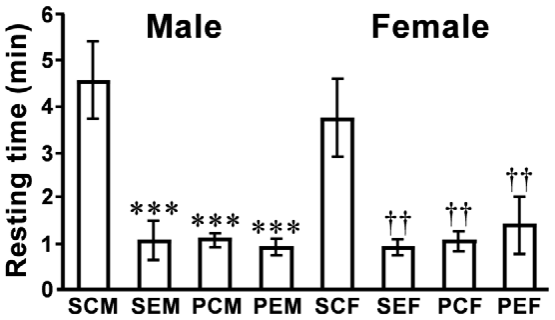
Effect of GnIH RNAi on the activity of white-crowned sparrows. Spontaneous resting time of control male (SCM), experimental male (SEM), control female (SCF), and experimental female (SEF) birds in 10 minutes; and the resting time of control male (PCM), experimental male (PEM), control female (PCF), and experimental female (PEF) birds in 10 minutes averaged from their responses to four male song playbacks. The columns and the vertical lines represent the mean ± SEM (n = 8). ***, *P*<0.001 vs SCM, ††, *P*<0.01 vs SCF by one-way ANOVA followed by Fisher’s PLSD.

GnIH gene silencing significantly increased spontaneous activities in both male and female birds, measured by recording their resting time [Fig. 3; Male, F = 12.9, *P* = 1.8×10^−5^ by one-way ANOVA, *P* = 2.7×10^−5^ SCM (spontaneous control male) vs. SEM (spontaneous experimental male) by Fisher’s PLSD; Female, F = 5.85, *P* = 0.0031 by one-way ANOVA, *P* = 0.0010 SCF (spontaneous control female) vs. SEF (spontaneous experimental female) by Fisher’s PLSD]. GnIH RNAi made the birds very active, similar to the behavior of birds when they were challenged by novel male song playbacks [Fig. 3; Male, *P* = 2.8×10^−5^ SCM vs. PCM (control male with song playback), *P* = 1.5×10^−5^, SCM vs. PEM (experimental male with song playback); Female, *P* = 0.0017 SCF vs. PCF (control female with song playback), *P* = 0.0055 SCF vs. PEF (experimental female with song playback) by Fisher’s PLSD]. There was no statistical difference in the time spent for feeding and the numbers of hopping and pointing between the groups (Figure S1).

### Effect of GnIH RNAi on the vocalizations of male and female birds

**Figure 4:**
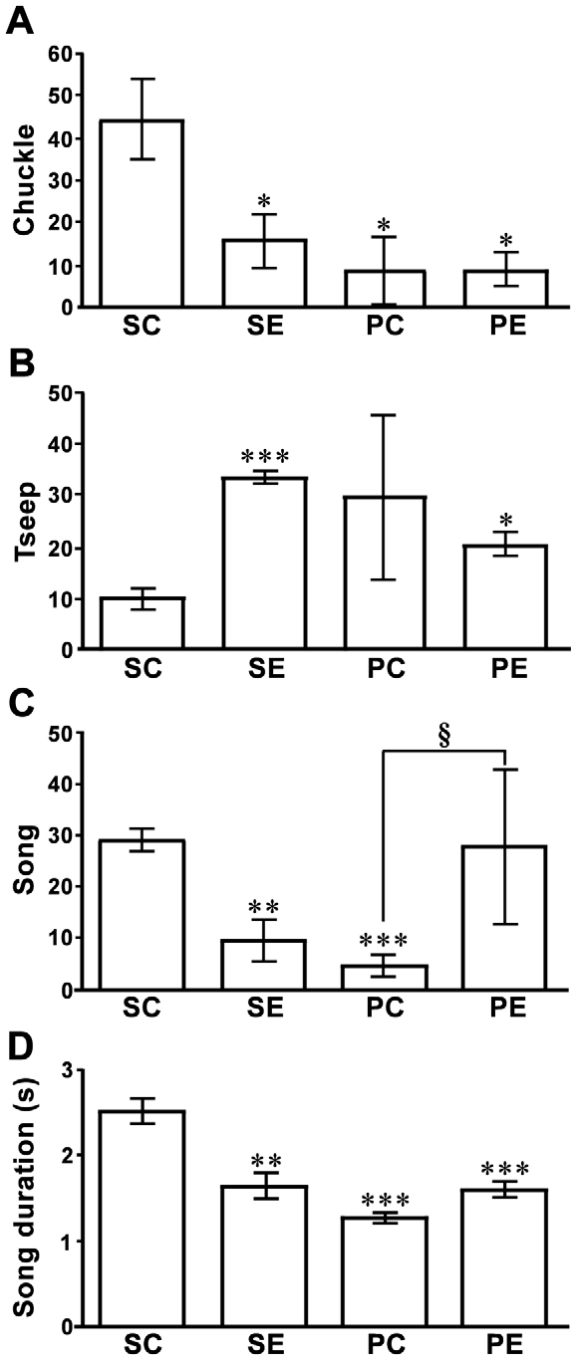
Effect of GnIH RNAi on the vocalizations of white-crowned sparrows. ***A,*** Total number of spontaneous chuckle in control (SC) and experimental (SE) birds in 10 minutes (n = 4), and total number of chuckle in control (PC) and experimental (PE) birds in 10 minutes in response to male song playbacks (n = 4). *, *P*<0.05 vs SC by one-way ANOVA followed by Fisher’s PLSD. ***B,*** Total number of spontaneous tseep in SC and SE birds in 10 minutes (n = 4), and total number of tseep in PC and PE birds in 10 minutes in response to male song playbacks (n = 4). *, *P*<0.05, ***, *P*<0.001 vs SC by one-way ANOVA followed by Fisher’s PLSD. ***C,*** Total number of spontaneous song in SC and SE birds in 10 minutes (n = 4), and total number of song in PC and PE birds in 10 minutes in response to male song playbacks (n = 4). **, *P*<0.01, ***, *P*<0.001 vs SC by one-way ANOVA followed by Fisher’s PLSD. §, *P*<0.05, PC vs PE, Kruskal-Wallis test followed by Mann-Whitney U-test. ***D,*** Spontaneous song duration of control (SC) and experimental (SE) birds in 30 minutes recordings (n = 4); and song duration of control (PC) and experimental (PE) birds in response to male song playbacks (n = 4). The columns and the vertical lines represent the mean ± SEM. **, *P*<0.01, ***, *P*<0.001 vs SC by one-way ANOVA followed by Fisher’s PLSD.

Spontaneous chuckle was significantly reduced by GnIH RNAi treatment as also seen in birds challenged by male song playbacks [Fig. 4A, F = 5.5, *P* = 0.027 by one-way ANOVA, *P* = 0.045 SC (spontaneous control birds) vs. SE (spontaneous experimental birds), *P* = 0.028 SC vs. PC (control birds with song playback), *P* = 0.013 SC vs. PE (experimental birds with song playback) by Fisher’s PLSD]. On the contrary, spontaneous tseep was significantly increased in GnIH gene silenced birds (Fig. 4B, F = 37, *P* = 4.5×10^−5^ by one-way ANOVA, *P* = 6.1×10^−5^ SC vs. SE, *P* = 0.014, SC vs. PE by Fisher’s PLSD), suggesting that GnIH gene silencing induces alertness or arousal in birds. Spontaneous song was significantly reduced by GnIH RNAi treatment as also seen in birds challenged by male song playbacks (Fig. 4C, F = 19, *P* = 6.2×10^−4^ by one-way ANOVA, *P* = 0.0064 SC vs. SE, *P* = 2.1×10^−4^ SC vs. PC by Fisher’s PLSD). However, song in response to playback of novel male songs was significantly increased by GnIH RNAi treatment (Fig. 4C, *P* = 0.013 by Kruskal-Wallis test, U = 16, Z = −2.3, *P* = 0.021 PC vs. PE by two-tailed Mann-Whitney U test), suggesting that GnIH gene silencing induces aggressiveness when there is a territorial challenge. We also measured the song duration to characterize the quality of the songs. Song duration of spontaneous song was reduced by GnIH RNAi treatment as was also seen in birds challenged by male song playbacks (Fig. 4D, F = 20.3, *P* = 3.5×10^−5^ by one-way ANOVA, *P* = 0.0016 SC vs. SE, *P*  = 5.9×10^−5^ SC vs. PC, *P* = 6.7××10^−4^ SC vs. PE by Fisher’s PLSD).

### Analysis of GnIH neuronal system in the brain

**Figure 5:**
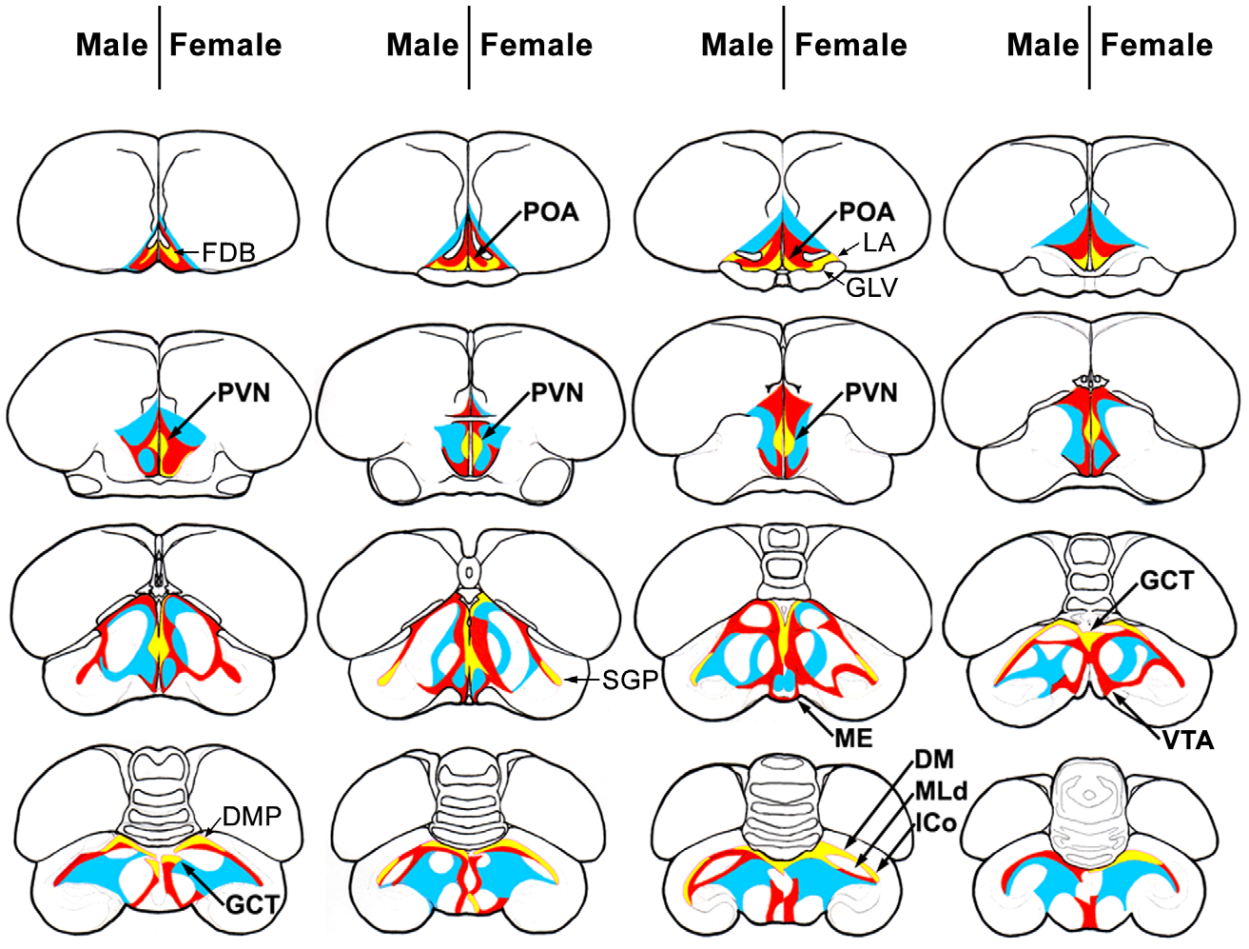
Schematic representations of white-crowned sparrow brain sections showing the locations and densities of GnIH fibers. Different colors indicate the densities of GnIH-ir fibers (yellow: high, red: moderate, blue: low). The number of dots of the immunoreactive beaded fibers was counted in 200 µm square in the center of each brain nucleus. High: over 75; moderate: 50 to 74; low: 25 to 49. The left-hand side of each brain summarizes the results of male control birds (M), whereas the right-hand side summarizes the results of female control birds (F). FDB: fasciculus diagonalis Brocae; POA: nucleus preopticus anterioris; GLV: the nucleus geniculatus lateralis, the pars ventralis; LA: the nucleus lateralis anterior thalami; PVN: nucleus paraventricularis; SGP: substantia grisea et fibrosa periventricularis; ME: median eminence; CGT: substantia grisea centralis; VTA: ventral tegmental area; DMP: nucleus dorsomedialis posterior thalami; DM: nucleus dorsalis medialis; MLd: nucleus mesencephalicus lateralis, pars dorsalis; ICo: lateral nucleus intercollicularis.

**Figure 6:**
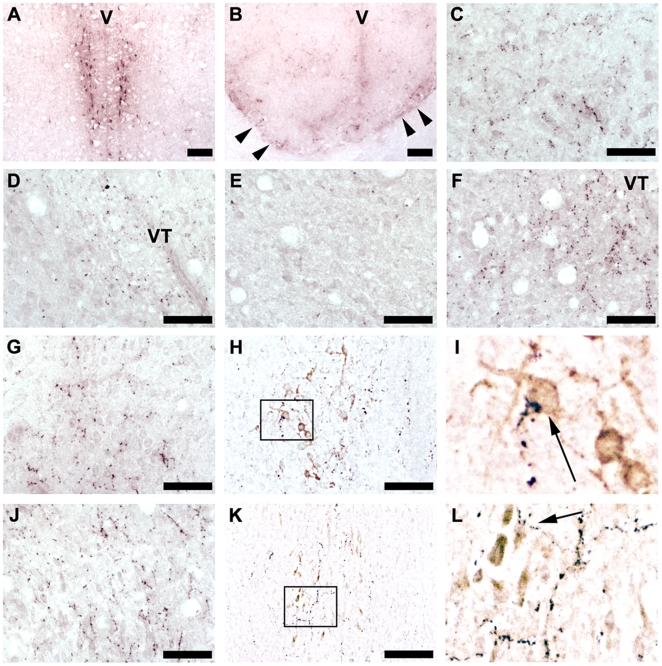
Expression of GnIH cell bodies and fibers in the white-crowned sparrow brain. ***A***, Expression of GnIH-immunoreactive (-ir) cells in the nucleus paraventricularis (PVN). V: Third ventricle. Bar, 100 µm. ***B***, GnIH-ir fibers in the median eminence. Arrowheads indicate GnIH-ir fibers in the external layer of the median eminence. Bar, 100 µm. ***C***, GnIH-ir fibers in the ventral tegmental area (VTA). Bar, 50 µm. ***D***, GnIH-ir fibers in the nucleus dorsalis medialis (DM). VT: Ventriculus tecti mesencephali. Bar, 50 µm. ***E***, GnIH-ir fibers in the nucleus mesencephalicus lateralis, pars dorsalis (MLd). Bar, 50 µm. ***F***, GnIH-ir fibers in the nucleus intercollicularis (ICo). Bar, 50 µm. ***G***, GnIH-ir fibers in the nucleus preopticus anterioris (POA). Bar, 50 µm. ***H***, GnRH-ir neurons (brown) with GnIH-ir fibers (purple) in the POA. Bar, 50 µm. ***I***, Higher magnification of the highlighted area in ***H*** showing a GnIH-ir fiber in the close proximity to a GnRH-I neuron. ***J***, GnIH-ir fibers in the substantia grisea centralis (CGT). Bar, 50 µm. ***K***, GnRH-ir neurons with GnIH-ir fibers in the GCT. Bar, 50 µm. ***L***, Higher magnification of the highlighted area in ***K*** showing a GnIH-ir fiber in the close proximity to a GnRH-II neuron.

We further analyzed the GnIH neuronal system in the brain that possibly regulated the activities of birds. GnIH-immunoreactive (-ir) cells were clustered in the PVN of hypothalamus (Fig. 5, 6A). Although GnIH-ir fibers were emanating to various parts of the brain (Fig. 5), abundant GnIH-ir fibers were observed in the fasciculus diagonalis Brocae (FDB), the nucleus preopticus anterioris (POA, Fig. 6G), the nucleus geniculatus lateralis, the pars ventralis (GLV), the nucleus lateralis anterior thalami (LA), PVN, the substantia grisea et fibrosa periventricularis (SGP), the median eminence (ME, Fig. 6B), the substantia grisea centralis (GCT, Fig. 6J), and the ventral tegmental area (VTA, Fig. 6C) (Fig. 5). GnIH-ir fibers were also observed in the nucleus dorsomedialis posterior thalami (DMP), and the brain nuclei related to vocalization and audition, such as the nucleus dorsalis medialis (DM, Fig. 6D) [28], the nucleus mesencephalicus lateralis, pars dorsalis (MLd, Fig. 6E) [28], [29], and the lateral nucleus intercollicularis (ICo, Fig. 6F) [28], [29] (Fig. 5). Neurons in VTA are thought to regulate the activities of the song control nuclei [30], [31]. It is known that two subtypes of GnRH [32], [33], GnRH-I [34]–[38] and GnRH-II [39] are expressed in the avian brain [40]. GnRH-I, GnRH-II neurons are present in the POA and GCT, respectively [4]. Because abundant GnIH-ir fibers were expressed in these brain nuclei, we also investigated the interactions of GnIH-ir fibers with GnRH-I, GnRH-II neurons. Close appositions of GnIH-ir neuronal fiber terminal like structures were frequently observed on GnRH-I (Fig. 6H, 6I) and GnRH-II (Fig. 6K, 6L) neurons in male and female birds. The overall pattern of GnIH fiber distribution was similar between male and female birds (Fig. 5).

### Effect of GnIH RNAi on the density of GnIH-ir fibers in the brain

**Figure 7:**
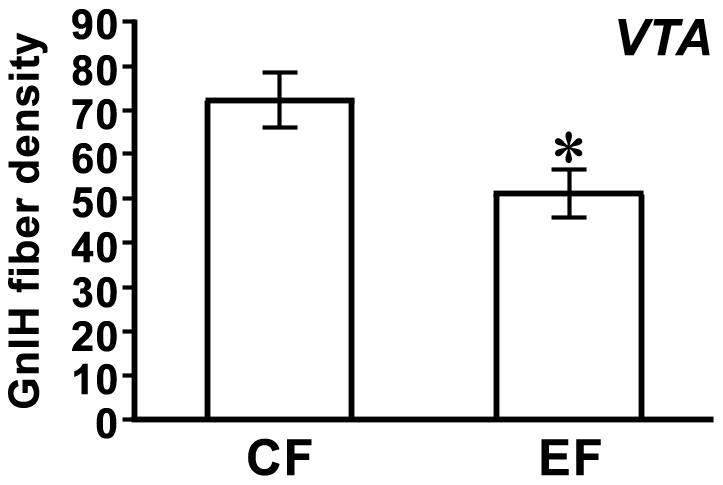
Effect of GnIH RNAi on the density of GnIH-ir fibers in the female ventral tegmental area. The number of dots of the beaded GnIH-ir fibers was counted in 200 µm square in the center of the ventral tegmental area (VTA) of control female (CF) and experimental female (EF) birds. The columns and the vertical lines represent the mean ± SEM (n = 8). *, *P*<0.05 vs. CF by two-tailed Student’s *t*-test.

We investigated the effect of GnIH RNAi on the density of GnIH-ir fibers in POA, GCT, VTA, DM, MLd and ICo in male and female birds. Significant reduction of GnIH-ir fiber density was observed in the VTA of female birds [Fig. 7, *t* = 2.6, *P* = 0.022 CF (control female) vs. EF (experimental female) by two-tailed Student’s *t*-test]. GnIH-ir fiber density tended to decrease in the MLd and ICo in female birds (MLd, *P* = 0.056, CF = 24.9±5.12, EF = 12.3±3.26; ICo, *P* = 0.052, CF = 130±9.43, EF = 101±9.75; two-tailed Student’s *t*-test).

### Relationships between the GnIH neuronal system and the activities of birds

**Figure 8:**
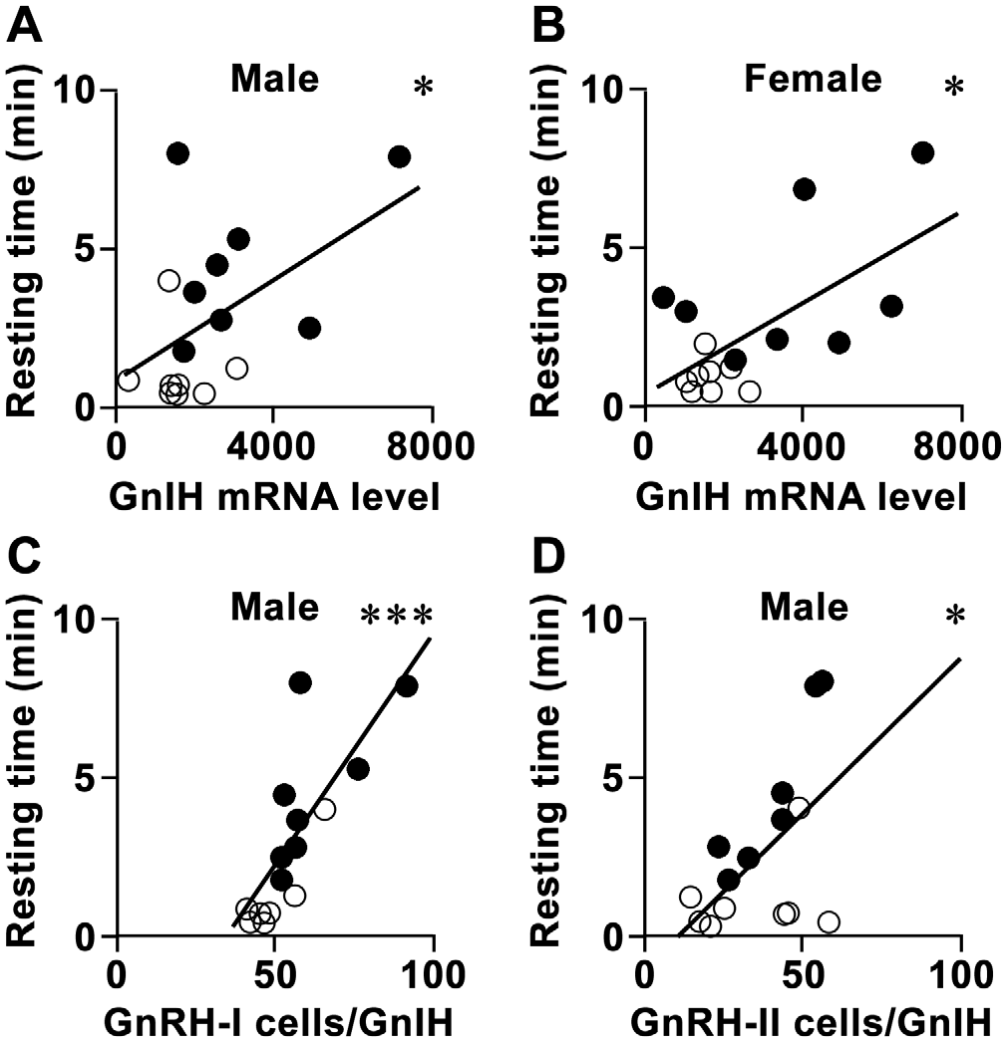
Relationships between the resting time and the GnIH neuronal system. ***A***, Relationship between the resting time and GnIH mRNA level in the PVN of male birds. Closed circles indicate the results of control birds, whereas open circles indicate the results of experimental birds. N = 15, R = 0.51, *, *P*<0.05 by two-sided Pearson’s correlation test. ***B***, Relationship between the resting time and GnIH mRNA level in the PVN of female birds. N = 15, R = 0.63, *, *P*<0.05 by two-sided Pearson’s correlation test. ***C***, Relationship between the resting time and the number of GnRH-I neurons with close appositions of GnIH-ir neuronal fiber terminal like structures in male birds. N = 15, R = 0.78, ***, *P*<0.001 by two-sided Pearson’s correlation test. ***D***, Relationship between the resting time and the number of GnRH-II neurons with close appositions of GnIH-ir neuronal fiber terminal like structures in male birds. N = 15, R = 0.55, *, *P*<0.05 by two-sided Pearson’s correlation test.

To understand the brain mechanism regulating the activity of birds by the GnIH neuronal system, we analyzed the relationships between the resting time and the GnIH neuronal system of birds (Fig. 8). GnIH mRNA levels (Fig. 2B) were positively correlated with the resting time of male (Fig. 8A, n = 15, R = 0.51, *P* = 0.049 by two-sided Pearson’s correlation test) and female (Fig. 8B, n = 15, R = 0.63, *P* = 0.011 by two-sided Pearson’s correlation test) birds. The number of GnRH-I neurons with close appositions of GnIH-ir neuronal fiber terminal like structures produced a strong correlation with the resting time of male birds (Fig. 8C, n = 15, R = 0.78, *P* = 5.8×10^−4^ by two-sided Pearson’s correlation test). The number of GnRH-II neurons with close appositions of GnIH-ir neuronal fiber terminal like structures in male birds was also positively correlated with their resting time (Fig. 8D, n = 15, R = 0.55, *P* = 0.032 by two-sided Pearson’s correlation test). The numbers of GnRH-I or GnRH-II neurons were not affected by GnIH RNAi treatment (Figure S2). GnIH-ir fiber densities in the VTA tended to correlate with the resting time of male birds (n = 15, R = 0.48, *P* = 0.072 by two-sided Pearson’s correlation test). On the contrary, no correlation was detected between the resting time of male birds and plasma testosterone concentration (Figure S3, n = 14, R = 0.41, *P* = 0.14 by two-sided Pearson’s correlation test).

## Discussion

This study was conducted to investigate the role of GnIH neurons in the regulation of behavior in the white-crowned sparrow, a highly social vertebrate species. GnIH neuron projections to various parts of the brain suggest direct regulations of behavior by GnIH [4], [5], [7]. Previous studies have shown that central administration of GnIH inhibits sexual behavior in birds and mammals [8], [13]. We accordingly tested the effect of central administration of siRNA against GnIH precursor mRNA in this study. There are reports that direct administration of a naked siRNA into the central nervous system can effectively silence the target mRNA expression in the brain resulting in changes in physiology and behavior [41]–[46]. We directly administered siRNA against GnIH precursor mRNA into the third ventricle in the proximity of PVN where GnIH precursor mRNA is solely expressed. Accordingly, it was also shown in the present study that central administration of GnIH siRNA can significantly reduce GnIH precursor mRNA and GnIH expression and change the activities of birds, thus validating the effect of direct administration of siRNA into the brain in birds.

GnIH RNAi stimulated general activities of male and female birds as if they were challenged by a novel intruder (analogous to territorial challenge) judging from their resting time. On the other hand, GnIH RNAi did not stimulate or inhibit specific behavior, such as feeding, preening, drinking, hopping or pointing. GnIH RNAi also stimulated agonistic vocalization, and induced song production in male birds in response to novel male song playbacks. An animal with a greater degree of CNS arousal (*1*) shows greater responsiveness to sensory stimuli in all sensory modalities; (*2*) emits more motor activity; and (*3*) is more reactive emotionally [17]. Here we show evidence that GnIH RNAi can induce generalized arousal because GnIH RNAi (*1*) stimulated song production in male birds in response to playbacks of novel male songs, (*2*) increased locomotor activity, and (*3*) stimulated tseep and song that may express emotions of songbirds.

A previous study indicated that central administration of GnIH to the third ventricle of female white-crowned sparrows inhibits copulation solicitation [8]. Accordingly, we hypothesized that reduction of GnIH expression by RNAi may positively regulate sexual behavior, in contrast to the GnIH administration. There is a report showing that mice selected for high levels of generalized arousal also show high levels of sexual arousal [47]. Because GnIH RNAi induced generalized arousal in this study, there was a possibility that GnIH RNAi also induced sexual arousal. Cages of male or female birds were placed alternately so that they could interact with birds of the opposite sex. However, GnIH RNAi did not induce spontaneous male courtship song to surrounding female birds. Instead, male song was stimulated in GnIH RNAi birds when the birds were challenged by novel male song playbacks that simulated territorial challenge. Male white-crowned sparrows in the wild sing shorter songs in territorial defense [48]. In our study, GnIH RNAi reduced song duration similar to those of control birds that were challenged by novel song playbacks. Accordingly, it is likely that GnIH RNAi induces agonistic territorial behavior in male birds rather than female-directed sexual behavior.

GnIH gene downregulation significantly increased spontaneous activities of birds. If the suppression of GnIH neuronal activities in the brain can induce arousal, there may be a significant relationship between the activities of birds and GnIH neuronal fiber distributions in specific brain areas. We investigated this by analyzing the relationships between GnIH neuronal fiber distributions and the activities of the birds. We found that the numbers of GnRH-I and -II neurons with close appositions of GnIH-ir neuronal fiber terminal-like structures were positively correlated with the resting time of male birds. GnIH neuronal fibers terminate on GnRH-I and GnRH-II neurons [18], and these neurons express GnIH receptor mRNA in songbirds [4]. Accordingly, it is possible that GnIH inhibits arousal by decreasing the activities of GnRH-I and GnRH-II neurons. Central administration of GnRH-II can stimulate female sexual behaviors in birds [19]. The direct action of GnRH-I neurons on behaviors in birds is not clear, but had no effect on copulation solicitation in female white-crowned sparrows [19]. On the other hand, it is known that central administration of GnRH-I can stimulate female sexual behaviors in rats [49], [50]. Further study is required to investigate how GnIH neurons inhibit arousal by regulating GnRH neurons in birds and mammals, and their sex differences.

GnIH orthologs have been identified in the rhesus macaque [11] and humans [12]. Detailed investigation of GnIH-ir fiber distributions in the primate brain showed similar distribution of GnIH-ir fibers compared to birds, such as in the POA and GCT [11]. GnIH-ir fibers were also observed in close proximity to GnRH-I, and GnRH-II neurons in the POA and GCT, respectively [11], [12]. Accordingly, the involvement of this hypothalamic inhibitory neuropeptide system in the suppression of arousal by regulating GnRH neurons may be a conserved property in birds and mammals including primates.

GnIH-ir fibers were also observed in the VTA, and GnIH-ir fiber densities in the VTA tended to correlate with the activity of male birds. GnIH-ir fibers in the VTA may have suppressed the generalized arousal state of the CNS in female birds because densities of GnIH-ir fibers were significantly reduced. In males VTA neurons project to the song control nuclei such as area× [30] and HVC [31]. Accordingly, it is also possible that GnIH neurons modify the vocalization of birds by regulating specific cells in the VTA.

GnIH-ir fibers were further observed in the DM, MLd and lateral ICo in this study. Because DM is related to vocalizations, GnIH may modify them by acting on this nucleus. GnIH may also affect song perception, because MLd is a part of the ascending auditory pathway, the avian homolog of the central nucleus of the inferior colliculus in mammals. Recently, a similar distribution of GnIH-ir fibers in the auditory and vocalization control nuclei was described in the zebra finch brain [6].

It is hypothesized that interactions between neurons in the ventrolateral preoptic nucleus and various cell groups in the brainstem regulate sleep and arousal [51], and closely related neuropeptides named orexins [52] or hypocretins [53] stabilize the wakefulness of the animal [54], [55]. Orexin/hypocretin neurons are produced exclusively in the lateral hypothalamic area (LHA) and they project to the brainstem arousal system [51]. Because GnIH neurons also project to the brainstem neurons in mammals [11] there is a possibility that GnIH inhibits arousal by acting on the brainstem arousal system. On the other hand, it was recently shown that GnIH neurons also project to orexin/hypocretin neurons in sheep [56]. It is therefore possible that GnIH neurons inhibit arousal by inhibiting orexin/hypocretin neurons. Future study is required to determine the interactions of GnIH neurons and the brainstem arousal system and orexin/hypocretin neurons in the LHA.

To sum up the results, although GnIH mRNA levels were positively correlated with the resting time of both male and female birds, the correlation between the resting time and the interaction of GnRH and GnIH neurons was only clear in male birds. On the other hand, GnIH fiber density in the VTA was reduced only in the female but not in male birds. Accordingly, although the spontaneous resting time or the resting time by GnIH RNAi or novel male song playback treatments was similar between male and female birds, the brain system involved in the regulation of various behavior of male and female birds may be different between sexes.

Although all vertebrates undergo reproductive maturation at least once in their lifetime, it has also been clear that local environmental cues trigger actual onset of reproductive activity such as nesting, sexual behavior and ovulation. The data presented here suggest that GnIH suppression of arousal could be the mechanism preventing premature triggering of reproductive activity. Suppression of GnIH release by favorable environmental cues signaling conditions conducive for breeding is a novel, but now eminently testable, hypothesis that could have important implications for adjustment of breeding cycles in response to climate change.

## Supporting Information

Figure S1 **Effect of GnIH RNAi on various behaviors of white-crowned sparrows.** Spontaneous behaviors of control male (SCM), experimental male (SEM), control female (SCF), and experimental female (SEF) birds in 10 minutes; and the behaviors of control male (PCM), experimental male (PEM), control female (PCF), and experimental female (PEF) birds in 10 minutes averaged from their responses to four male song playbacks. The columns and the vertical lines represent the mean ± SEM (n = 8).
(TIF)

Figure S2 **Effect of GnIH RNAi on the number of GnRH-I and GnRH-II neurons.** ***A*** **,** The number of GnRH-I cells in control male (CM), control female (CF), experimental male (EM), and experimental female (EF) birds. ***B*** **,** The number of GnRH-II cells in CM, CF, EM, and EF birds. The columns and the vertical lines represent the mean ± SEM (n = 8).
(TIF)

Figure S3 **The relationship between the resting time and plasma testosterone concentration in male birds.** Closed circles indicate the results of control birds, whereas open circles indicate the results of experimental birds. N = 14, R = 0.41, *P* = 0.14 by two-sided Pearson’s correlation test.
(TIF)
