# Supplementary figures and images for: RNA Interference of Gonadotropin-Inhibitory Hormone Gene Induces Arousal in Songbirds
Source: PLoS One. 2012 Jan 18;7(1):e30202. doi: 10.1371/journal.pone.0030202 (PMC3261185; doi:10.1371/journal.pone.0030202)

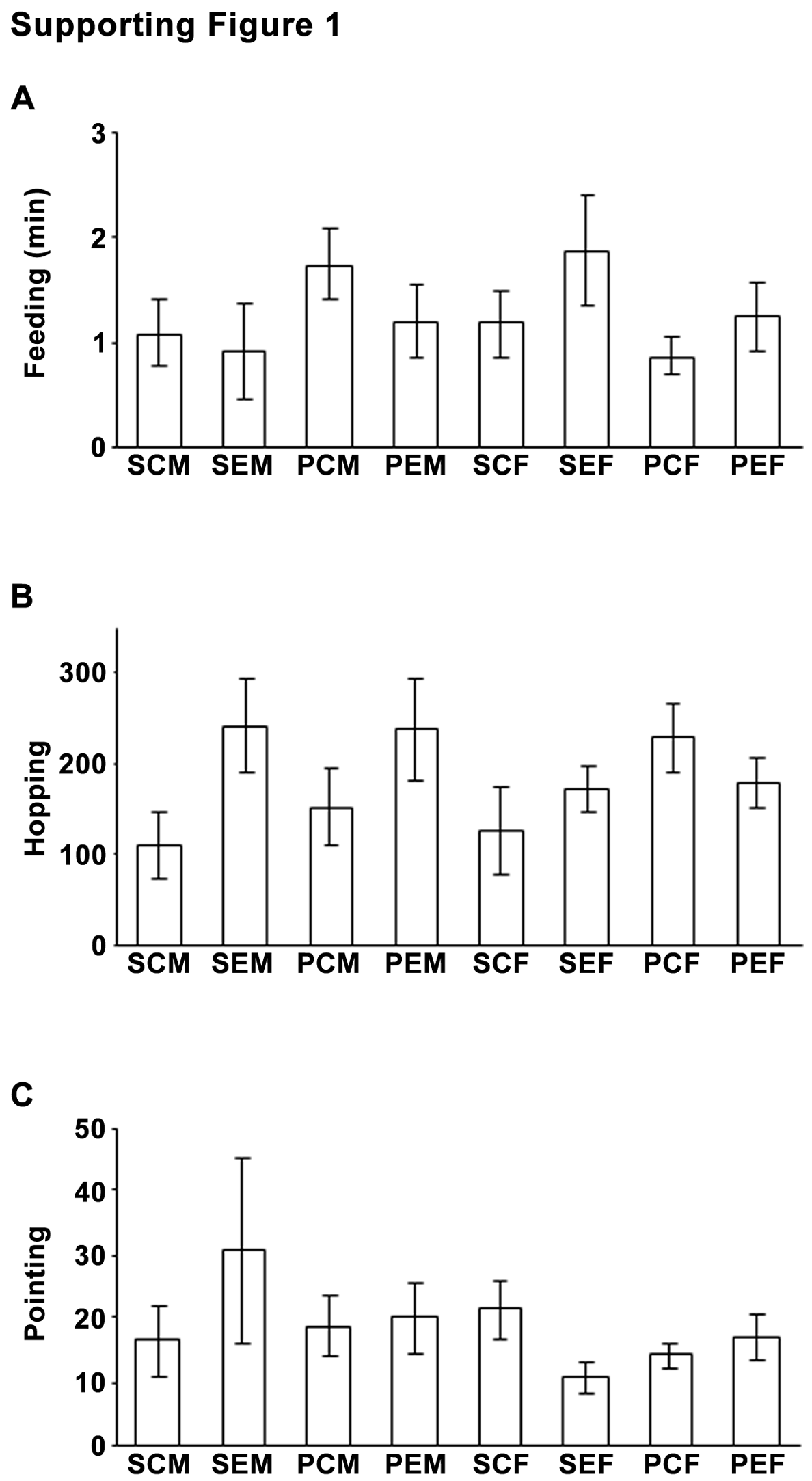

Supplement: Figure S1 — Effect of GnIH RNAi on various behaviors of white-crowned sparrows. Spontaneous behaviors of control male (SCM), experimental male (SEM), control female (SCF), and experimental female (SEF) birds in 10 minutes; and the behaviors of control male (PCM), experimental male (PEM), control female (PCF), and experimental female (PEF) birds in 10 minutes averaged from their responses to four male song playbacks. The columns and the vertical lines represent the mean ± SEM (n = 8). (TIF) [file pone.0030202.s001.tif]

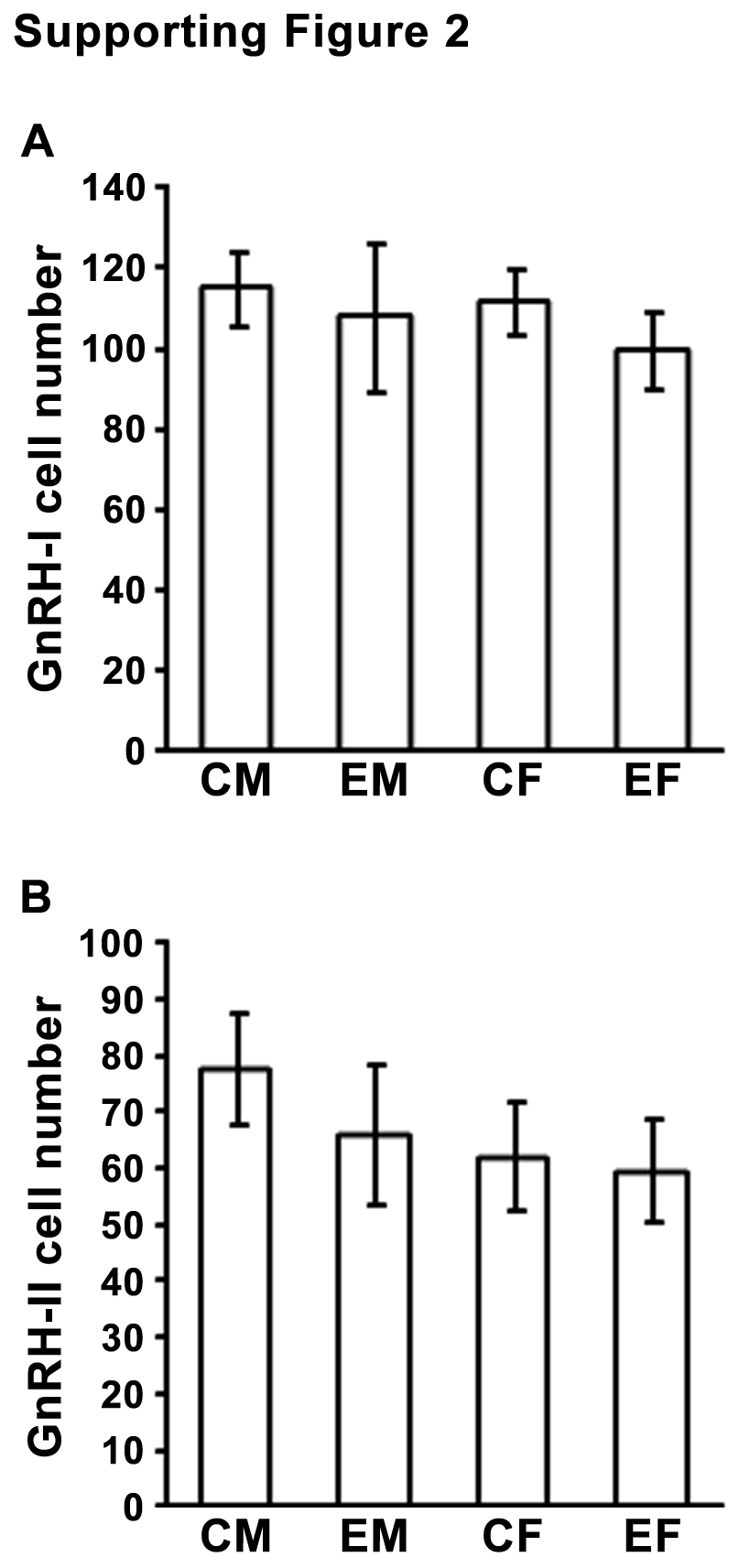

Supplement: Figure S2 — Effect of GnIH RNAi on the number of GnRH-I and GnRH-II neurons. A , The number of GnRH-I cells in control male (CM), control female (CF), experimental male (EM), and experimental female (EF) birds. B , The number of GnRH-II cells in CM, CF, EM, and EF birds. The columns and the vertical lines represent the mean ± SEM (n = 8). (TIF) [file pone.0030202.s002.tif]

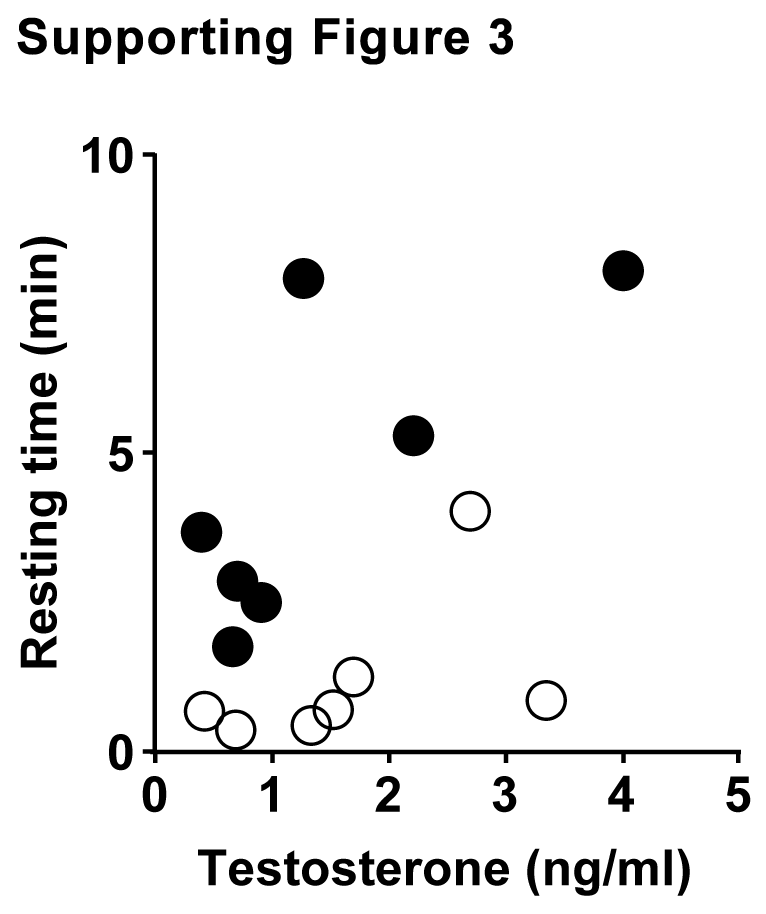

Supplement: Figure S3 — The relationship between the resting time and plasma testosterone concentration in male birds. Closed circles indicate the results of control birds, whereas open circles indicate the results of experimental birds. N = 14, R = 0.41, P = 0.14 by two-sided Pearson’s correlation test. (TIF) [file pone.0030202.s003.tif]
